# Inflammation is a key contributor to ovarian cancer cell seeding

**DOI:** 10.1038/s41598-018-30261-8

**Published:** 2018-08-17

**Authors:** Dongyu Jia, Yoshiko Nagaoka, Makoto Katsumata, Sandra Orsulic

**Affiliations:** 1 Women’s Cancer Program at the Samuel Oschin Comprehensive Cancer Institute, Cedars-Sinai Medical Center, Los Angeles, CA USA; 2 Department of Biomedical Sciences, Cedars-Sinai Medical Center, Los Angeles, CA USA; 3 Department of Obstetrics and Gynecology, David Geffen School of Medicine, University of California, Los Angeles, Los Angeles, CA USA; 4 Present Address: Department of Biology, Georgia Southern University, Statesboro, GA USA

## Abstract

The incidence of ovarian cancer dramatically increases in early menopause but the factors contributing to cancer onset are unclear. Most ovarian cancers originate in the fallopian tube with subsequent implantation of malignant cells into the ovary. However, the events and conditions that lead to cancer cell implantation are unknown. To quantify which conditions are conducive to the seeding of cancer cells in an immunocompetent mouse model, we surgically implanted mouse ovarian cancer cells into the oviducts of syngeneic mice and simulated conditions associated with ovulatory wound repair, incessant ovulation, ovarian surface scarring, and aging. We found that the dominant site of cancer cell seeding was not the ovary but the nearby surgical wound site, which was associated with a strong and persistent inflammatory reaction. Conditions in the ovary associated with inflammation, such as acute ovulatory wound repair, active healing of the scarred ovarian surface, and mouse aging, contributed to increased seeding of the cancer cells to the surgical wound site and tissues surrounding the ovary. Changes in the ovary not accompanied by inflammation, such as completed ovulatory cycles and fully-healed scars on the ovarian surface, did not contribute to increased cancer cell seeding. We conclude that inflammation is the most likely mechanism by which ovulation and postmenopausal events contribute to the increased risk of ovarian cancer.

## Introduction

Despite modern day cytoreductive surgical techniques and combination chemotherapies for high-grade ovarian cancer, five-year survival rates remain below 40%^1^. However, when found early, the survival rate dramatically rises to 90%^1,2^. Thus, the ability to detect ovarian cancer in its earliest stages is critical to a cure. It is increasingly accepted that high-grade ovarian cancers actually originate in the fallopian tube with malignant cells shedding to the adjacent ovary^3–7^. Since the bulk of the tumor typically forms in the ovary, rather than the fallopian tube, ovaries must play a significant role in the early stages of cancer development. Discovering which cellular and molecular processes promote and inhibit the seeding of malignant cells to the ovary could facilitate the development of markers for early detection as well as the identification of rate-limiting events in the early stages of ovarian cancer development. If contextual molecular cues provided by the ovary are required for the clinical development of ovarian cancer, such molecules could serve as novel therapeutic targets to prevent cancer progression in the early stages, when cures are more viable.

Epithelial ovarian cancer is predominantly a disease of postmenopausal women^8^. Many theories of postmenopausal onset of ovarian cancer have been proposed, including incessant ovulation and inflammation, hormonal changes, reduced immunity, increased cell senescence, and uncontrolled production of reactive oxygen species^9–13^. Epidemiologic data consistently show that the risk of ovarian cancer increases with the number of ovulatory cycles^14–16^, indicating that ovulation plays a significant role in ovarian cancer etiology. However, the peak incidence of menopause occurs at age 51, while the peak incidence of invasive epithelial ovarian cancer occurs at age 63^1^. Thus, most women develop ovarian cancer years after their last ovulatory cycle. Currently, it is unknown which conditions in the ovary promote tumor growth but the fact that more than 80% of ovarian cancer cases occur after menopause suggests that the events associated with menopause and aging are major contributing factors^8^.

During the postmenopausal years, ovarian follicles are largely depleted and much of the remaining ovary is reduced to a collagenous scar tissue^17^. If the microenvironment of postmenopausal ovaries is conducive to the implantation of cancer cells, simulating postmenopausal conditions should result in more cancer cell deposits in the ovary. A better understanding of ovarian cancer pathogenesis, specifically the role of the early postmenopausal ovarian microenvironment in supporting the seeding and survival of malignant cells in the ovary, is necessary to develop strategies for ovarian cancer prevention and detection. Experiments in mice provide a convenient system in which both the effect and the outcome of specific conditions can be examined and quantified. Previously, we used a mouse model to study events associated with ovulation and ovulatory wound repair, including epithelial cell entrapment and the formation of epithelial inclusion cysts^18^. Here, we extended those studies by simulating various postmenopausal conditions in mice and quantifying cancer cell deposits for each condition. The goal of the study was to determine whether conditions associated with ovulation and aging increase the spread of cancer cells from the oviduct to the ovary. To account for a possible role of the immune system in ovarian cancer cell seeding, we used an immunocompetent FVB mouse model with syngeneic ovarian cancer cell aggregates implanted into the fallopian tube. Our data show that premenopausal and postmenopausal conditions contribute to increased cancer cell seeding only in the presence of an inflammatory reaction.

## Materials and Methods

### Cancer cell line

The FVB-syngeneic mouse ovarian cancer cell line, BR, was engineered with combinations of genetic alterations (p53-/-, Brca1-/-, myc, and Akt) as described^19^. We have shown that this ovarian cancer model recapitulates human serous histology, pattern of metastatic spread, and response to standard and targeted therapies^19–23^. The BR cells were subsequently transduced with luciferase lentiviral plasmid pLenti-CMVPuroLUC (Addgene, w168-1) to generate BR-luc cells.

### Preparation of cell aggregates

BR-luc cells were seeded at a density of 1 × 10^6^ cells per well in Costar ultra-low attachment 6-well plates (Corning). The cells were incubated with 3 ml DMEM media in 5% CO_2_ at 37 °C. After 2 days, culture media were collected in 15 ml conical tubes and cells were precipitated at 1000 rpm for 0.5 minutes. After two rounds of washing with phosphate buffered saline (PBS), large cell aggregates were separated into small aggregates by multiple pipetting through a 1 ml pipette tip.

### Injection of cell aggregates into oviducts

All procedures in mice were performed in accordance with the approved Cedars-Sinai IACUC protocol (IACUC5318). The procedures were performed in an AAALAC-accredited facility at Cedars-Sinai Medical Center. The surgical procedures were performed according to the method described for embryo transfer into the oviduct (Manipulating the Mouse Embryo: A Laboratory Manual, 3rd Edition, ISBN-978-087969591-0). Under the dissecting microscope, a small incision between the infundibulum and the ampulla of the oviduct (equivalent to human fallopian tube) was created using Vannas scissors (Supplementary Video 1). The transfer pipette loaded with cell aggregates in PBS was inserted into the incision with the tip pointing toward the ovary and approximately 200 cell aggregates in 2 μl volume were injected into each oviduct (Supplementary Video 1).

### Simulation of ovulatory and menopausal conditions

Mice were superovulated by intraperitoneal injection of pregnant mare serum (PMS) and human chorionic gonadotropin (hCG) as previously described^18^. In the control mice, PBS was injected instead of PMS and hCG. To generate scar tissue, bursa (a thin membrane covering the ovary in mice) was removed (Supplementary Video 2) and the ovarian surface was burned with a hand-held battery-powered cauterizer (Gemini Cautery System) (Supplementary Video 3).

### Quantification of cancer cell deposits

Mice were euthanized by CO_2_ asphyxiation followed by cervical dislocation prior to harvesting the ovaries and surrounding tissues. To quantify macroscopic tumors, dimensions (length, width, height) were measured by calipers. Tumor volume (mm^3^) was calculated using the equation V = (L × W × H)/2, where V is tumor volume, L is tumor length, W is tumor width, and H is tumor height. For the flat, superficial tumors that typically formed on the surgical wounds/scars, tumor area (mm^2^) was measured using the equation A = L × W, where A is tumor area size, L is tumor length, and W is tumor width. To quantify microscopic cancer cell deposits, the ovaries, oviducts, and surrounding fat tissues were fixed in formalin and embedded in paraffin. One 4 µm-thick section per sample was stained with hematoxylin and eosin (H&E) and evaluated under the light microscope for visible cancer cell deposits.

### Statistical analyses

The statistical analyses were performed using GraphPad Prism (version 6.0; GraphPad Software). Intergroup differences were assessed by the Student’s *t*-test.

### Data availability

No datasets were generated or analyzed during the current study.

## Results

Our ability to screen for early stage ovarian cancer is hampered by deficiencies in the understanding of the molecular and morphological steps involved in ovarian carcinogenesis. It is currently unknown why cancer cells in the fallopian tube have the propensity to migrate to the ovary where they tend to form a large tumor mass. To determine which ovarian conditions are most conducive to implantation of detached tubal cells, we simulated in mice conditions associated with ovulatory wound healing, incessant ovulation, atrophy/scarring, and aging.

### Inflammatory events associated with ovulatory wound repair contribute to increased cancer cell seeding to tissues surrounding the ovary but are not directly associated with the implantation of cancer cells to the ovary

**Figure 1:**
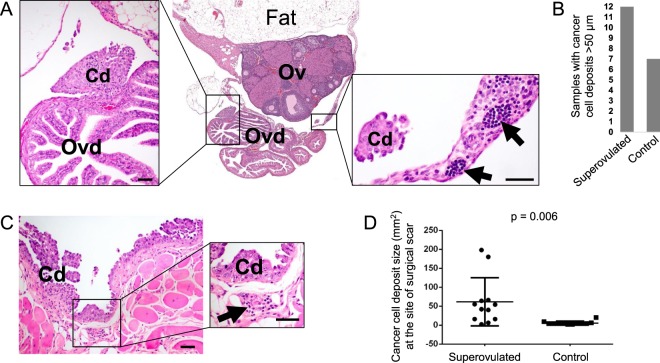
Assessing the effect of ovulatory wound repair on cancer cell seeding from the oviduct to the ovary and adjacent tissues. **(A)** Representative H&E-stained section of cancer cell deposits on the oviduct and ovary. Arrows indicate immune cell infiltrates. Size bars: 50 µm. Cd, cancer cell deposit; Ov, ovary; Ovd, oviduct. **(B)** Graph indicates the number of superovulated and control ovary/oviduct samples containing cancer cell deposits larger than 50 µm in diameter (out of 12 ovaries in each group). **(C)** H&E-stained section representing ‘carpeting’ of cancer cells along the surgical wound/scar site in the peritoneal wall. Arrow indicates an immune cell infiltrate. Size bars: 50 µm. Cd, cancer cell deposit. **(D)** Comparison of cancer cell deposit size at the surgical wound/scar site in superovulated and control mice.

To simulate cancer cell seeding and entrapment during ovulatory wound healing, superovulation was induced in 4 week-old female FVB mice by intraperitoneal injection of PMS and hCG hormones (superovulated group, N = 6) or PBS (control group, N = 6). This combination of hormones induces ovulation of a large number of follicles to form 10–30 acute ovulatory wounds within one ovulatory cycle^18^. Two days after hCG (or control PBS) injection, when ovulatory wound repair is at its peak^18^, cancer cell aggregates were bilaterally implanted into the mouse oviduct. Three weeks later, intraperitoneal tumor dissemination was evaluated by recording the presence of ascites and measurable tumor deposits inside of the peritoneal cavity. Macroscopically visible swelling was observed in 4/12 ovaries from the superovulated mice and in 0/12 ovaries from the control mice. Microscopic cancer cell deposits in the oviducts, ovaries, and surrounding fat were quantified by pathologic examination of H&E-stained sections under the 4x objective, and the presence of cancer cells was further verified under higher magnification (Fig. 1A). The deposits in tissues surrounding the ovary were frequently associated with immune cell infiltrates (Fig. 1A). Cancer cell deposits larger than 50 µm were present in tissues surrounding the ovary (oviduct, bursa, and space between the fat and ovarian surface) in 12/12 samples from the superovulated mice and in 7/12 samples from the control mice (Fig. 1B). However, neither group of mice exhibited cell deposits directly on the ovarian surface or as intraovarian inclusions. These results suggest that ovulatory wound healing is not directly associated with the implantation of cancer cells to the ovary. In both groups of mice, the largest cancer cell deposits presented as carpeting of the abdominal wall at the sites of surgical wounds/scars (Fig. 1C). The surgical wound/scar cancer cell deposits were frequently associated with immune cell infiltrates (Fig. 1C) and were significantly larger in superovulated mice than in control mice (Fig. 1D). Taken together, our results indicate that events associated with ovulatory wound healing contributed to increased seeding of cancer cells to the surgical site and tissues surrounding the ovary. The lack of cancer cell deposits attached to the ovarian surface indicates that re-epithelialization of the ovarian surface does not significantly contribute to cancer cell seeding. It is more likely that ovulatory events contributed to increased inflammatory infiltrates, which attracted cancer cells and/or supported their survival and expansion.

### Ovarian atrophy resulting from previous incessant ovulation is not associated with increased cancer cell seeding

To simulate cancer cell seeding in ovaries that endured repeated damage and repair due to multiple cycles of ovulation, six week-old female FVB mice were subjected to nine weeks of weekly intraperitoneal PMS and hCG hormone injections (repeatedly superovulated group, N = 7) or PBS injections (control group, N = 7). To mimic conditions in postmenopausal women whose ovaries have not been actively cycling for years, we waited 12 weeks after the last superovulation to implant BR-luc cell aggregates into the oviducts of the repeatedly superovulated and control mice. Eight weeks after cancer cell implantation, the tumor burden was evaluated in both groups. The majority of tumor deposits were found at the surgical wound/scar tissue, which was frequently fused with the adjacent fat and infiltrated with immune cells (data not shown). There were no macroscopically or microscopically visible cancer cell deposits on the ovaries and oviducts in either group of mice (data not shown). Thus, in the absence of acute inflammation, ovaries that have undergone repetitive superovulations do not appear to attract cancer cells any more than age-matched ovaries with a normal number of ovulatory cycles. One caveat to this experiment is that we did not achieve complete depletion of the oocytes pool despite nine cycles of superovulation, possibly because mice become unresponsive to hormone induction after reaching reproductive maturity^24^.

### Burn-induced scarring of the ovarian surface is associated with increased cancer cell seeding to the ovaries and surrounding tissues only in the presence of active scar wound healing

**Figure 2:**
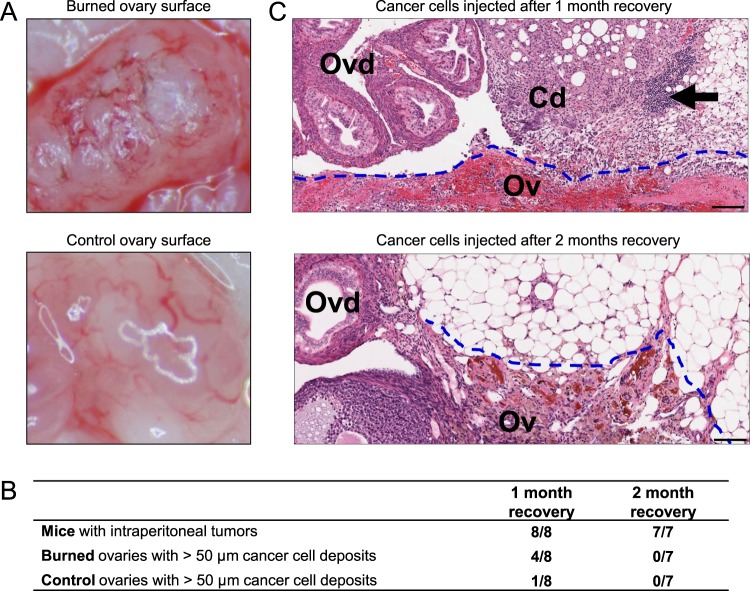
Assessing the effect of burn-induced ovary scarring on the seeding of intraperitoneally injected cancer cells. **(A)** Representative images of ovarian surface immediately after manipulation. Control ovaries were surgically released from the bursa while burned ovaries were first surgically released from the bursa, then superficially burned with a cauterizer. **(B)** Comparison of seeding efficiency of intraperitoneally injected cancer cells one or two months after surgical ovary manipulation. **(C)** Representative H&E-stained sections of cancer cell deposits on the ovaries and oviducts of mice that were injected one or two months after surgical ovary manipulation and euthanized four weeks later. Arrow indicates an immune cell infiltrate. Size bars: 100 µm. Cd, cancer cell deposit; Ov, ovary; Ovd, oviduct.

To simulate events associated with postmenopausal ovary atrophy and connective tissue scarring, burn-induced scars were generated on the ovarian surface of the six week-old female FVB mice. In each mouse, one ovary was surgically released from the ovarian bursa (Supplementary Video 2) and superficially burned with a cauterizer (Fig. 2A and Supplementary Video 3). The contralateral ovary was surgically released from the bursa but not burned (control ovary) (Fig. 2A). Mice were intraperitoneally injected with a single-cell suspension of BR-luc cells (~1 × 10^6^ cells) after one month recovery (N = 8) or two months recovery (N = 7). Four weeks after intraperitoneal cell injection, mice were euthanized for tumor burden quantification. Regardless of whether mice were intraperitoneally injected with cancer cells one month or two months after surgery, BR-luc cells formed multiple small tumor nodules on the mesothelial surfaces of the omentum, pancreas, diaphragm, spleen and abdominal lining; however, there were no visible tumor cell deposits on the surface of the burned or control ovaries. Therefore, we assessed microscopic cancer cell deposits in H&E-stained sections of ovaries/oviducts and adjacent fat. For the one month recovery group (N = 8), cancer cell deposits larger than 50 µm were present in the tissues surrounding the ovary (fat, oviduct, and bursa) in 4/8 burned ovaries and in 1/8 control ovaries (Fig. 2B). All ovaries that contained tumor deposits also had abundant immune cell infiltrates (Fig. 2C). For the two months recovery group (N = 7), none of the ovary sections contained cancer cell deposits (Fig. 2B). Although burn-induced scars on the ovarian surface were detectable two months later, the scars were no longer associated with abundant immune cell infiltrates (Fig. 2C). These results suggest that burn-induced scars attract cancer cells but only in the presence of inflammation.

### Events associated with aging contribute to increased cancer cell seeding to the ovaries and surrounding tissues

**Figure 3:**
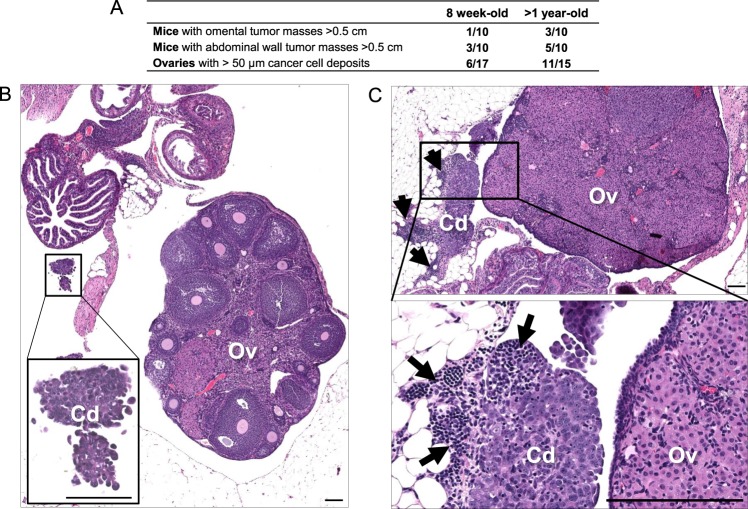
Assessing the effect of aging on cancer cell seeding from the oviduct to the ovary and adjacent tissues. **(A)** Comparison of seeding efficiency of cancer cells implanted into the oviducts of young (8 weeks) and aged (>1 year) mice. In H&E-stained sections, several ovaries were excluded from the analysis because they were either missing from the slide section or the tissue was insufficient for evaluation. **(B**,**C)** Representative H&E-stained section of cancer cell deposits on ovaries and oviducts four weeks after surgical implantation of cancer cells into the oviducts of (**B**) 8-week-old mice and **(C)** >1-year-old mice. Arrow indicates an immune cell infiltrate. Size bars: 100 µm. Cd, cancer cell deposit; Ov, ovary.

BR-luc cancer cell aggregates were bilaterally implanted into the oviducts of eight week-old (N = 10) and greater than one year-old (age range 14–19 months; N = 10) female FVB mice. Mice were euthanized for analysis four weeks after cancer cell implantation. Both groups of mice developed multiple intraperitoneal metastases with the largest tumor masses present on the omentum and abdominal wall. Omental and abdominal wall masses were more frequent in aged mice (Fig. 3A). Three of the aged mice also exhibited unilateral or bilateral uterine horn hyperplasia (data not shown). H&E-stained sections showed that the ovaries from young mice contained multiple follicles in different phases of maturation (data not shown), while the ovaries from old mice were devoid of follicles (Fig. 3B). Microscopic examination of the ovaries and adjacent tissues (oviduct, bursa, and adjacent fat) revealed that tumor cell deposits were more frequent in aged mice (Fig. 3A), which also contained more abundant immune cell infiltrates (Fig. 3B). These results suggest that ovaries from aged mice are more conducive to cancer cell seeding than ovaries from young mice.

## Discussion

A poor understanding of the initiating events in ovarian cancer has significantly hampered our efforts towards early ovarian cancer detection and prevention. Most early stage cancers in the tubal fimbria are associated with a dominant mass in the ovary, indicating that the ovarian microenvironment is essential for tumor growth. However, conditions that promote cancer cell seeding and growth in the ovary are still unknown. Recently, Yang-Hartwich and colleagues used a mouse xenograft model to test the role of ovulatory wound repair in the migration of cancer cells from the injection site in the uterus toward the ovary^25^. Consistent with epidemiologic data that increased ovulation is strongly associated with ovarian cancer^15,16^, they showed that superovulation in mice enhances the migration and adhesion of malignant cells to the ovary and that this attraction is mediated through the release of cytokines/chemokines from the surface wound created by oocyte release^25^. Using a syngeneic immunocompetent mouse model with cancer cells surgically implanted into the oviduct, we confirmed that superovulation contributes to ovarian cancer cell seeding. Tumor cell deposits were accompanied by immune infiltrates, indicating that ovulation-induced inflammation may play an important role in cancer cell seeding. It is possible that the inflammatory reaction is the only factor that contributes to increased cancer cell seeding because the largest cancer cell deposits typically formed in the abdominal wall along surgical wounds, which were associated with extensive immune infiltrates. It appears that the wounded surface of the superovulated ovary did not play a direct role in cancer cell attraction as there were no cancer cells attached to the ovarian surface epithelium or inside the ovarian stroma. The importance of the inflammatory reaction, rather than the damaged ovarian surface in cancer cell seeding, was illustrated by the next two sets of experiments in which we repeatedly wounded the ovarian surface by multiple rounds of superovulation or burned the ovarian surface to induce scarring. The wounded/scarred ovarian surface proved to be attractive to cancer cells only if the wounds were ‘fresh’. If ovarian wounds/scars were allowed to recover for two months, cancer cells were no longer attracted to the ovarian surface but were still attracted to other sites in the peritoneal cavity where inflammation persisted. It is well established that aging is characterized by subclinical, chronic inflammation^26^. Consistent with multiple studies showing that the overall proinflammatory status in older mice is associated with increased tumor burden^27^, our results show that oviductal implantation of cancer cells in aged mice resulted in increased tumor burden throughout the peritoneal cavity.

Our finding that surgical wounds in mice attract cancer cells is consistent with an observation in clinical practice that wound trauma in patients is associated with cancer recurrence^28,29^. It has been shown that an early peak of breast cancer recurrence is due to surgery-driven intervention^30^. The exact reasons for surgery-related cancer attraction are not fully understood but possible factors include surgery-related acute wound healing process, inflammation, and activation of dormant cancer cells by surgery-driven growth factors^31–33^. If inflammation is a key factor in cancer cell seeding, what are the contributions of other factors strongly associated with increased cancer incidence, such as ovulation, oocyte depletion and atrophy, and aging? Our data in a mouse model are consistent with the concept that most of the factors implicated in ovarian cancer incidence converge on inflammation as a common denominator. One successful path to ovarian cancer prevention has been controlling factors that induce inflammation, such as the use of oral contraceptives to suppress ovulation^34^. Epidemiologic data show that aspirin and other nonsteroidal anti-inflammatory drugs (NSAIDs) can be beneficial in the prevention of multiple cancers, including ovarian^35,36^. Although factors associated with the increased risk of ovarian cancer, such as aging and menopause cannot be prevented, the risk can be reduced by suppressing inflammation. The results of our study in a mouse model confirm previous results that inflammation is a key factor in promoting ovarian cancer cell seeding. An understanding of the mechanisms by which inflammation plays a role in the early stages ovarian cancer will be necessary for effective ovarian cancer prevention.

## Electronic supplementary material

Transfer of cancer cell aggregates into the oviduct

Bursa removal

Ovarian surface scarring
